# Development of a Sensor to Measure Physician Consultation Times

**DOI:** 10.3390/s19245359

**Published:** 2019-12-05

**Authors:** Roman Gabl, Florian Stummer

**Affiliations:** 1 School of Engineering, Institute for Energy Systems, FloWave Ocean Energy Research Facility, The University of Edinburgh, Max Born Crescent, Edinburgh EH9 3BF, UK; 2 Deanery of Molecular, Genetic and Population Health Sciences, Usher Institute, The University of Edinburgh, Old Medical School, Teviot Place, Edinburgh EH8 9AG, UK

**Keywords:** people counting, bi-directional, motion detection, time of flight sensor

## Abstract

The duration of patient–physician contact is an important factor for the optimisation of treatment processes in healthcare systems. Available methods can be labour-intensive and the quality is, in many cases, poor. A part of this research project is to develop a sensor system, which allows the detection of people passing through a door, including the direction. For this purpose, two time of flight sensors are combined with a door sensor and a motion detection sensor (for redundancy) on one single side of the door frame. The period between two single measurements could be reduced to 50 ms, which allows the measurement of walking speed up to 2 ms${}^{-1}$. The accuracy of the time stamp for each event is less than one second and ensures a precise documentation of the consultation time. This paper presents the development of the sensor system, the miniaturisation of the installation and first measurement results, as well as the measurement’s concept of quality analysis, including multiple door applications. In future steps, the sensor system will be deployed at different medical practices to determine the exact duration of the patient–physician interaction over a longer time period.

## 1. Introduction

Demographic changes around the world bring new challenges to healthcare systems. People now live longer and their healthy years increase, especially in higher- and middle-income countries. However, a longer life expectancy can also mean that individuals have to live longer with illness [1]. The treatment of chronic illnesses and accompanying comorbidities is a primary healthcare system issue, as it leads to continuous public health monitoring through healthcare professionals and an increase of patient–physician contacts.

In 2017, Irving et al. [2] presented the results of an international survey on variations in primary care physicians’ consultation times. The results show a wide range of consultation periods: from 48 s in Bangladesh, through 15 min in Australia, to 22.5 min in Sweden. Eighteen countries, which hold more than 50 percent of the world’s population, needed less than 5 min to treat a patient. However, the main finding was the lack of continuous data collection. A majority of countries were not able to provide adequate data on measurement methods, the person measuring time, the study design, the general duration in min, the number of consultations, and the quality of data sets. The paper at hand introduces actual standard measurement methods and presents a possible new solution for direct and automated gross patient–physician interaction duration measurement. This new sensor-led collection method aims to exclude biased data collection and the feared “Hawthorne”-effect to a certain extent.

The primarily used measurement methods can be categorised as (a) self-reported, (b) video, (c) audio, (d) stopwatch, (e) SMS (short message service), (f) International Network for the Rational Use of Drugs (INRUD), and (g) calculation-centred. Each method is introduced and discussed below:

(a) Self-reported means that a member of the healthcare staff (mostly the treating physician) answered a survey or provided a collection of duration times. These self-reported figures can, however, be categorised as biased.
(b) Video means that a video recording of the activities in the treatment room were evaluated. This approach needs the patient’s recruitment and consent and is highly problematic regarding the General Data Protection Regulation (GDPR).
(c) Audio recordings are used to verify the duration of patient–physician interactions; however, similarly to the video method, audio recordings are problematic regarding GDPR.
(d) As part of the stopwatch method, a member of the healthcare staff (mostly the treating physician) timed the consultation period. This method is more reliable, as the net results can be evaluated. However, a comparison to gross results is not possible.
(e) A short message (SMS) is sent by a member of the healthcare staff or the physician to a certain phone number, deriving the consultation duration time from the received message.
(f) World Health Organisation/International Network for the Rational Use of Drugs (WHO/INRUD) means using data provided by the drug use indicators for health facilities, which contain a set of patient care indicators including the average consultation time. The number is evaluated by “dividing the total time for a series of consultations by the number of consultations”.
(g) Calculation means that certain time parameters were collected and average consultation duration times were derived. Most of the data sets used to do the calculations cannot be verified.

Irving et al. [2] found that the quality of studies using these methods were good in 40%, poor in 36%, and fair in 24%, leaving a ratio of 40% to 60% with regard to trustworthiness (good) versus dubiousness (poor/fair).

Knowing how long a patient–physician consultation lasts is not only a matter of improving the patient’s journey, but also a business administration factor. Taking the project management triangle (time, costs, scope, and quality) into account, time is the only factor that can neither be regained nor changed. However, costs can be derived from time. Scope and quality are functions of cost and time. A direct, automated time measurement method that excludes biases as far as possible, therefore allows a more substantial and sustaining documentation of treatment processes and the development of lean organisational systems for business administration, human resource departments, and healthcare staff interaction.

The paper presents a novel sensor system to automatically measure the interaction time between patient and physician in a single and multi-room configuration. Especially the latter requires a documentation of the time period individuals take to pass through a door and in which direction they are headed. Existing methods are often either strongly dependent on possibly biased, sometimes imprecise self-reporting, or they are problematic in regard to GDPR (video and/or audio recordings), and are also likely to be very labour-intensive. The novel sensors system aims to overcome those weaknesses, and allows the combination of the individual treatment period with anonymous patient data to verify the provided services, and additionally develop optimised operation methods in medical practices. The development of the proposed solution is summarised and discussed. Exemplary results are presented and a long term deployment of five sensors is currently ongoing.

## 2. Materials and Methods

### 2.1. Overview and Objectives

Sensors that count and track people are commercially available and deliver a wide range of applications in airports, museums, libraries, or any other type of (public) buildings. Possible applications are crowd management, transport systems, heating as well as air conditioning, and optimisation of evacuation planning [3]. Significant user groups are retailers and shopping centres. These can deploy available products to track the behaviour of costumers in different zones, identify specific targeted groups (children, families, and adults) and also combine those data with further available sales information. This helps to allocate staff and optimise marketing tools. An advanced application such as this requires stereo/video capturing, covering a wider area and specific analysis methods [4,5,6]. Li et al. [7] added a near-infrared stereo camera system for application in low light levels. Consequently, this method is available for nearly every condition but raises significant privacy issues.

The deployment of such a sensor system in a medical practice includes specific restrictions and ethical considerations. Patients and the content of the consultation with the physician need to stay fully anonymous. Each analysis based on video, which would be used in the previously mentioned commercially available solutions, can be stored and is subject to a potentially unauthorised access. Nevertheless, a video system can be used under specific conditions. An example is presented by Mousavi et al. [8], who recorded an operating room. Based on a manual analysis, he tracked how long and why the door was opened in relation to the different stages of the operation. The main objective of the presented development of a novel sensor system is to reduce risks regarding GDPR as well as the potential risk of abuse. The aim is to reduce these concerns in order to increase the patient’s as well as the physician’s trust and acceptance.

Hence, the instrumentation should only be capable of recording specific data needed for the research project. This restriction has to be included in the design and should not depend on filters, processing, or storing. Consequently, all video surveillance solutions are excluded and the choice of commercially available solutions are limited to light barrier. Various available products also include detection of direction (leaving or entering the door), but all found solutions are limited regarding the time resolutions. The internal data processing in the device sums up the detection and only adds up values for a time period of typically 15 min. This is absolutely sufficient for the above mentioned applications but is not enough for the detailed analysis of the time the patient interacts with the physician. Those interactions are typically in the range of minutes [2]. An extended search and the contact with various manufacturers and service providers brought the authors to the conclusion that no product allows access to the raw data or the needed resolution time. Consequently, the development of a specific instrumentation is required. An additional benefit is that it can be custom-made for the specific needs of the research project.

Door sensors, based on a magnetic contact, can deliver a basic evaluation of the time in which the patient or the physician enters the room. Each closing and opening of the door can be captured with a time stamp. However, there is no specification regarding the number of people passing through a door at once or whether they are entering or leaving a room. This results in an uncertainty and makes it hard to analyse the captured data. The deployment of a single beam switch improves the situation significantly and allows for counting of the number of individuals entering or leaving. A double beam approach is needed to determine the direction of motion as it is used in commercially available products. Alternative methods would have to face the motion direction, which can potentially distract from the movement of the person through the door. Bi-directional detection and the limitation to the door frame are key objectives for the development.

In addition to the mentioned requirements concerning ethical aspects, as well as the time resolution (a few seconds), the installation should be straightforward, unobtrusive, and not limited to specific conditions. This includes the requirement to contain the entire instrumentation in one unit and the elimination of a mirror, a reflector, or a (light) source on the other side of the door. The utilisation and combination of standardised components allow the minimisation of costs and ensure the durability of the system. A redundant documentation based on different methods helps to validate the results and reduces uncertainties. Furthermore, both temporary observation (a few days to weeks) and also the potential for a long time observation over months should be given. The developed sensor combination meets all those objectives and based on the presented analysis method an objective documentation of the duration of patient and physician interaction can be delivered.

### 2.2. Combination of the Sensors

**Figure 1:**
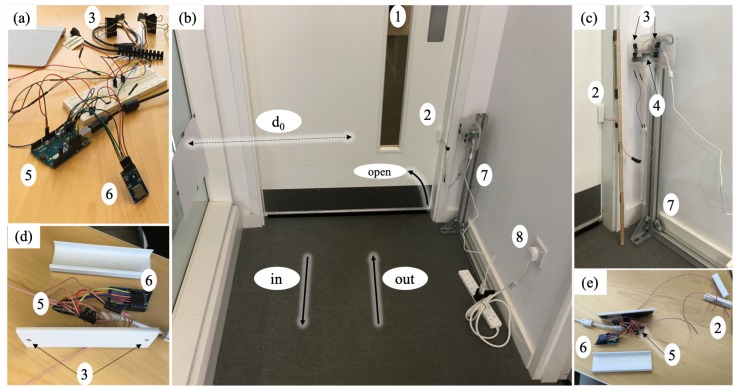
Development of the sensor system (**a**) desk version; (**b**,**c**) prototype installation to test the concept; (**d**,**e**) final design. Door (1), door sensor (2), time of flight (3), motion detection (4), Arduino (5), Micro SD card module (6), support structure (7), and power supply (8).

Based on the objectives (Section 2.1) a combination of three different sensors was chosen for the final design: (a) magnetic door sensor (DS), (b) one additional motion detection (MD) sensor (RCWL 0516), and (c) two time of flight (ToF) sensors (VL53L0X). All three different components are available from various suppliers and in a wide range of options (size, colour, and connections). The first tests were conducted with an Arduino Uno (Figure 1a), which has been replaced by an Arduino Nano for the final version due to the smaller dimensions (Figure 1d,e).

The door sensor is a magnetic surface contact. For the presented application a product was chosen, which is commonly used in alarm circuits (supplier CQR Security). This guarantees a high reliability and durability. It would also include a tamper alarm, which is unused. The operation distance between the switch and the magnet housing is dependant on the direction as well as the mounting position. It ranges from 5 mm to 35 mm for non-ferrous surfaces and is reduced in combination with ferrous components (door frame or panel). This information is provided in the operation and installation instructions by the supplier [9]. The DS delivers the basic information of if the door is open or closed and the Arduino logs the time stamp as soon as a change in the status occurs.

Initially different passive infrared sensors (PIR) were tested, which also allowed sensitivity and delay adjustments. Those motion sensors are commonly used as a light switch and typically deliver an output signal as long as motion is detected (including a further delay). The exact limitation of the activation range was not easy to achieve and the sensor system did not trigger precisely. Issues in collecting precise data are encountered if a person just steps into the room and immediately walks out again or if a person passes by on the door. An approach based on a line observation is more sensible and precise in comparison to a comparable big region, in which the sensor is the trigger. Consequently, PIR sensors were discarded in an early design state. Nevertheless, one single radar module RCWL 0516 [10] is still used in the presented instrumentation. This sensor detects motion with comparable accuracy to a PIR system but works through plastic. Hence, it can be very easy added to the systems and allows an additional independent motion detection as validation for the other systems.

Both previously mentioned methods are not capable of detecting the direction of motion. Therefore a delay between two lines (vertical planes) has to be measured. Huang et al. [11] showed a successful installation of a bi-directional system, which provides additional inputs for the building automation system. Therefore, infrared sources are mounted on one side of the door and the detector is mounted on the other side. An exact alignment is needed as well as devices on both sides of the door. Alternatively, the emitter and detector can be installed on one side and only a reflector is added opposite to the instrument. The previously mentioned objectives of the research include that the installation effort should be reduced. Different range finding devices are available, which allow the measurement of the distance without a specific arrangement on the target. As part of the discovery phase, commercially available small ultrasonic sensors and infrared distance meters were investigated. They were tested to determine if they are suitable to work in a pair and in close range. The investigated lower price devices did not deliver the needed quality nor reliability and those techniques were not further studied.

Far better results could be reached with a time of flight (ToF) distance sensor, which is typically used as a scanning device [12,13,14,15] rang finder [16,17,18] and gesture detection sensor [19]. The used VL53L0X module is capable of capturing distances up to a maximum range of 2 m with a very high accuracy. This specific device includes a Class 1 laser, which is integrated with the collector on the chip. The beam is invisible to the human eye and the spreading is comparatively small [20]. The communication with the Arduino is based on the I2C (Inter-Integrated Circuit) interface. For the current application, two devices are installed in parallel. This requires that as part of every power up the XSHUT pin (Rest or interrupt the communication) is activated to change the default address of the device. Two parallel distance measurements are captured, which allow us to identify the direction of the motion. Further details are presented in Section 3.1.

The motion detector as well as the two ToF sensors allow a clear identification, even when the door is kept open—if, for example, the physician switches between two rooms (Section 3.2). Under regular conditions, the combination of all three different types of sensors delivers an exact recording of the door state, an additional motion detection and two distances, which allows us to determine the motion direction of the person passing through the door.

### 2.3. Prototype Development

The development of the sensor system was done in three phases. For the initial desk version (Figure 1a), different sensor types and combinations were tested. Based on this, it was decided to use the final combination of sensors, which are installed (Section 2.2). Furthermore, the Arduino code was developed and tested (Section 3.1).

In a second step, a realistic configuration was built up and tested. Figure 1 b,c shows the set-up at one door at FloWave Ocean Energy Research Facility (University of Edinburgh). It is a fire door with a door closer, which connects the main office with the wave tank area. A piece of paper is used to limit the distance measurement of the ToF sensors on the opposing side as there is a big window. This construction provides a high flexibility of heights for the ToF sensors and the location is ideal to deploy a generously designed box. The presented results in Section 3 are captured with this set-up.

**Figure 2:**
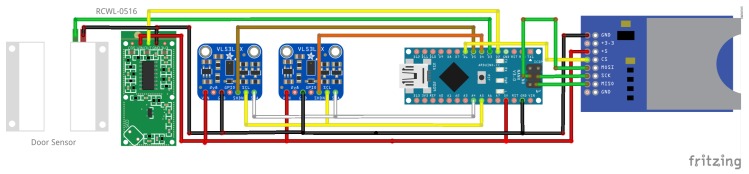
Shematic representation of the circuit including the Arduino Nano: power (red), ground (black) and all further colours mark connections to input/output pins.

Finally, an additional step in the development is made to further miniaturise the instrumentation (Figure 1). A vital part of this is the change from an Arduino Uno SMD R3 to a Nano (HiLetgo Mini USB Nano V3.0 ATmega328P CH340G), which has nearly the same capability but is far smaller in size. Figure 2 shows the circuit and how the sensors are connected to the Arduino. In the final design, the magnetic door contact remains as a separate unit and is connected via cable to the main unit. Thus, an independent installation as well as an optimal usage of the available space in the door frame is possible. It was noticed that the distance from the door to a wall socket is in the range of a few meters for all the physicians’ offices, where the sensor will be deployed in the next project phase. Hence, the power supply based on batteries was considered, however a direct power supply is preferred. This decision was made to secure a long term deployment of the system.

Different cases and boxes were investigated to enclose the system. The best fit and usability could be gained with a cable trunking as shown in Figure 1d,e. This provides protection of the electrical parts and a good accessibility. The distance between the two holes for the ToF sensor is 100 mm and all sensors are mounted to the cover piece. The needed cable feed-through can be included in the other part, which is attached to the door frame with double sided adhesive tape. This back part can be easily replaced and custom-made for each specific location. The dimensions are 38 mm by 16 mm and 120 mm in length. Both ends are closed with caps and can further be sealed with tape. Only a weak extraction-proof could be included in the casing and the cables have to be further secured with tape.

The captured data is stored for each individual sensor in one txt-file on a Micro SD card, which is included in the case. A wide range of different applications successfully included a real-time transmission of the measurement data ranging from traffic systems [21], displacement sensors of piles and columns [22], or weather stations [23]. For the current research project, reliable storage is more important than immediate transmission hence the analyses are conducted after the test. This also gives the opportunity to eventually combine the findings with anonymous treatment categories, which include factors like a patient’s age, gender, and main diagnosis. Consequently, a transmission of the data is not needed but is considered for future applications, hence the device also allows for remote management. The simultaneous observation of more than one door requires a synchronisation of several installed sensor systems. This is conducted as part of the installation and the possible time offset is acceptable.

## 3. Results

### 3.1. Single Door

**Figure 3:**
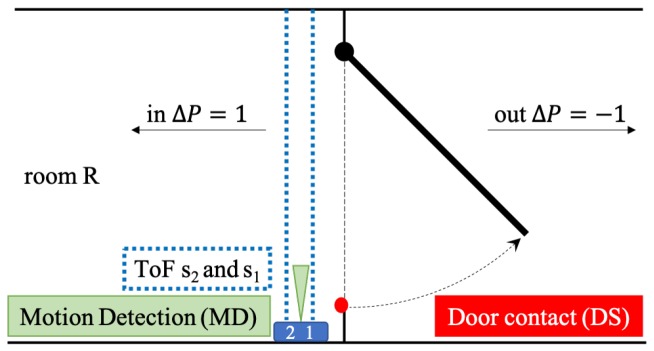
Exemplary sketch of the set-up of sensors and definition of the motion direction.

In this first section, the data capturing of the sensor system is described in general as well as the processing for one door. Figure 3 presents the exemplary set-up, which is similar to the one presented in Figure 1b. For the following consideration, it is assumed that the door solely gives access to one single room, in which the time of the interaction between physician and patient has to be measured. Therefore, the full data set is captured and the analysis is conducted as a post processing. Section 3.2 expands this to further doors and more than one room.

The processing tasks done on the Arduino are tried to keep as low as possible. This allows to go through the loops efficiently and to reduce the time step between two readings. Status changes of the motion detector (MD) as well as the door sensors (DS) are simplified to 0 and 1. This coded information is written in separate files including a time stamp. In this process, the time values for changes of the MD and DS are rounded to full seconds.

A different approach is implemented for the time of flight (ToF) sensors. In an initial step the distance between the sensors and the opposing wall is adapted. This allows us to define a location-specific criterion (it includes the measurements of both sensors) to trigger the recording of the two sensors. Each device measures the distance between the sensor and the next object individually, and the results is only stored when the criterion is fulfilled. Finally, the analysis method for the ToF data is presented.

The first 10 s after the initialisation of the Arduino (power is switched on) are used to adapt the code to the specific circumstances of the deployment location. An initial distance $d_{0}$ is defined in the code (typically 900 mm for the door width) and is adapted to the specific locations as follows:

(1) $$d_{j+1}=\left(d_{j}+s_{1}+s_{2}\right)/3\;\;\mathrm{with}\;d_{j}=d_{0}\;\mathrm{for}\;j=1.$$

Based on Equation (1), the measured distance of each sensor $s_{1}$ and $s_{2}$ is average with the distance to define the suitable distance $d_{0}$, which is set equal to $d_{j}$ at the end of the 10 s. The index ${}_{j}$ indicates one loop and each step of this iteration is documented in a separate text file. For the test deployment shown in Figure 1b the distance $d_{0}$ was 1110 mm. After this adaptation, the results of both ToF sensors are written in a further file only if the following criterion is fulfilled:

(2) $$s_{1}+s_{2}<d_{0}\cdot 2-200\;\mathrm{mm}.$$

This allows us to limit the file size by not recording the ToF sensor data if nothing happens. The 200 mm are based on the first tests and allow us to introduce a certain threshold before the values are written into the file so that the file size is not unnecessarily increased. Each line further includes the time in milliseconds. A typical time difference between two measurements is close to 50 ms (milliseconds). It can vary depending what else has to be done in the specific loop (for example the documentation of a change at the MD or DS). Theoretically, a walking speed of up to 2 m s${}^{-1}$ (=10 cm/50 ms) or 7.2 km h${}^{-1}$ can be captured. This is in an acceptable range [24,25,26,27,28], especially as the operation of the door reduces the walking speed.

The durability of the measurement system was proven with a continuous deployment over three weeks under comparable high traffic (approximately 10 people working in the office). The detected errors could be clearly associated with deliberate behaviour of colleagues. In a future step of the research project, the sensor system will be installed in different healthcare settings and tested under those specific conditions. Each installation will initially be paired with a further validation by observation (using the stopwatch method).

As part of the post processing, the file is imported into Matlab and the difference between both measured values is calculated:

(3) $$\Delta s=s_{2}-s_{1}.$$

**Figure 4:**
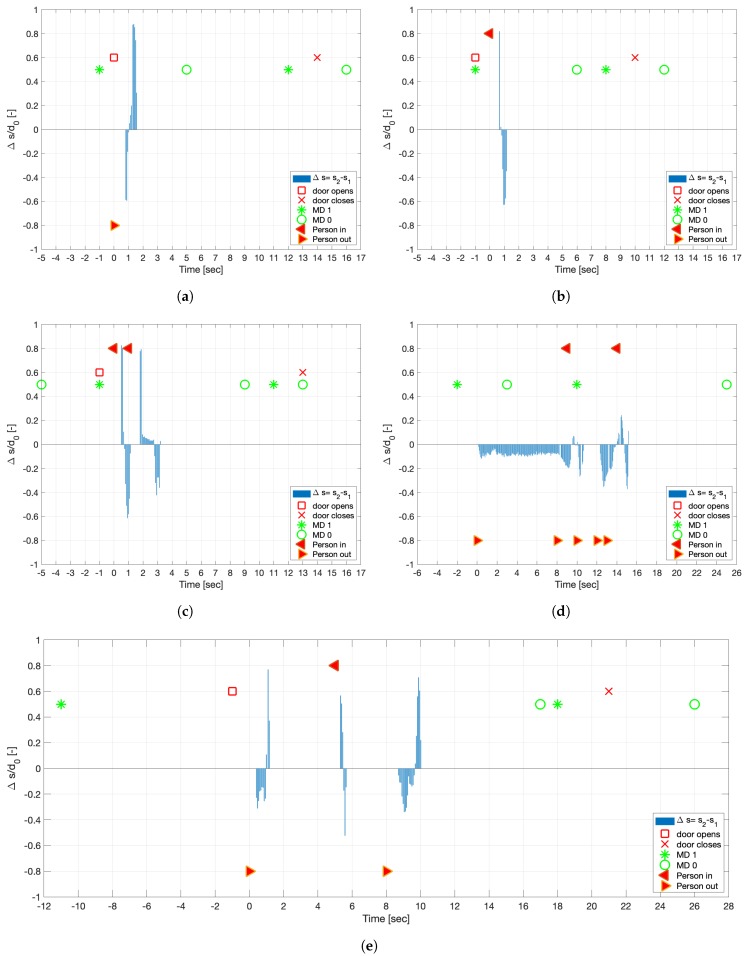
Exemplary result for different events. Results of MD and DS as well as the direction arrow are plotted with a constant offset (0.5, 0.6, and ± 0.8) for a better visualisation. (**a**) Outgoing event. (**b**) Incoming event. (**c**) Detection of two person entering the room. (**d**) Error detection caused by waiting person. (**e**) Outgoing person opens the door, which keeps open for an incoming and following outgoing event—MD stays up as long as motion happens and reacts again caused by the closing door.

Significant for the analysis of the direction is the algebraic sign of the Δs value. Figure 4 presents exemplary single events of only one person passing (a and b) as well as more complex events (c to e). In those cases, Δs was standardised by the adapted distance $d_{0}$, but this could be further extracted with a sign function. The presented time was set individually to 0 for the first found result based on the ToF sensor. This allows a direct comparison of the duration of the event. Depending on the speed of the person, the first two to five values of Δs define the direction. Further quality controls are possible, based on the availability of the full measurements of both sensors. An obvious check is to look for the zero crossing of Δs and the following changed algebraic sign when a person leaves the sensor area. Errors caused by person standing in front of the sensor can be detected and have to be either manually corrected or when it happens more, the algorithm can adapt. Passing of more than one person can also be detected as presented in Figure 4c, which can also be in different directions (Figure 4e).

The conducted test showed a very high reliability of this measurement concept and especially of the ToF sensors. Based on this experience, the hierarchy of the analysis is chosen as following: (1) ToF sensor as main indicator and (2) door contact as well as (3) motion detector to further validate the event detection.

The first event detection is based on the analysis of the ToF sensors. The time stamp of the first trigger measurement is stored in a vector $t_{i}$ as well as $\Delta P_{i}$ for the direction (values ± 1). The index *i* represents the number of events in the total observation time. A new event is defined, when the previous documented value is more than $\Delta t_{event}$ apart (typically 150 milliseconds, depending on the specific location). Those events are filters in a first step based on the difference between each event. Therefore, the time difference is calculated based on $\Delta t=t_{i-1}-t_{i}$. All events in which Δt is smaller a location specific threshold $\Delta t_{multiPers}$ (for example 2 s) is interpreted as multiple persons. The comparison between the two events presented in Figure 4c,d show potential problems. Those examples represent a real event and an error measurement caused by a person standing in front of the sensor. In the current version of the analysis algorithm such multi events are simplified and analysed assuming that up to three people moving close to each other go through the door shortly after each other. The event presented in Figure 4d was flagged for an additional check and had to be interpreted separately. The exact analysis method will be further developed based on the initial tests under real conditions, which will not contain such a slow automatic door closer. An additional value $\Delta t_{assistant}$ can be introduced, which allows to filter short-term stays of assisting personnel, additional physicians, or a patient’s attendants.

Those filtered events are further checked for plausibility. Firstly, the door has to be opened or be still open in a time window of $\pm \;\Delta t_{door}$. Furthermore should the motion detector indicate a movement, however, this is not mandatory hence an error in this sensor is comparably more likely. Based on this, an actual event with a moving person is stated and a ΔP with ± the number of moving persons—depending on the direction shown in Figure 3—defined. After those checks, the value of $t_{i}$ is similar to the other sensors rounded to full seconds.

The exemplary results in Figure 4 also clearly show that the closing door also indicates the motion sensor (door is closed by an automatic closer). An additional plausibility check is conducted for all door opening events, which are not connected to a motion detection based on the ToF sensors in the previous step. Those are manually checked. Further combinations of results and the conclusions are presented in Table 1.

The full data set is further split into separate days and the number of people in the room *R* is calculated by adding up the vector ΔP after each event. An obvious control is to find cases in which *R* is smaller than 0. Such an error can be caused for example by the following occurrence: Two persons enter the room together side by side (due to the support of a walking impediment) or one person enters directly behind the other (wheelchair). In those cases, the sensor system cannot detect a clear difference from a single (slow walking) person. When they leave separately, which will be correctly detected, such a problem can occur. The solution is to check the previous event manually or refine the algorithm depending on the specific location of the sensor. It is further advisable to match the total number of patient–physician interactions with the records of the day. The involved physicians are also asked to document unexpected circumstances, which could influence the measurements. They further note down the time and if they are currently talking to a patient or not (in a range of 1 or 2 h). This allows validation of the record of the sensors, as well as, in extreme cases, a reset of *R*.

### 3.2. Multi Door and Room Set-Up

**Figure 5:**
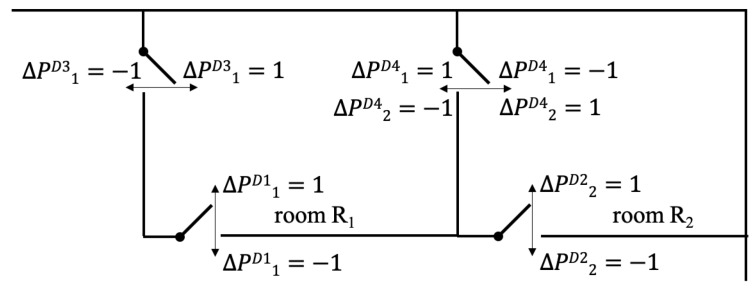
Example of a multiroom set-up. ΔP indicates the changes of the number of people in the room *R*.

In the previous section only the scenario of one single door into one room was covered. If there are two doors, obviously an additional sensor is needed. The more interesting case is a multi room set-up, as it is shown for example in Figure 5. This allows either to host two physicians or to give the opportunity of one physician switching between two rooms. In this case, the door D4 connects both rooms and does not necessarily have to be closed. If the person leaves one room, he or she enters the other room. The calculation can be either done for each event, which includes changing the length of the vectors, or based on a fixed time resolution. In the latter case, the vectors *R* and ΔP for each door are initially zero vector. The specific values of the detected event are added to the entry of the vector closest to $t_{i}$. The evaluation for each time step can be calculated as following:

(4) $$\begin{array}{c} R_{1,t+1}=R_{1,t}+\Delta P_{1}^{D1}+\Delta P_{1}^{D3}+\Delta P_{1}^{D4} \end{array}$$

(5) $$\begin{array}{c} R_{2,t+1}=R_{2,t}+\Delta P_{1}^{D2}+\Delta P_{2}^{D4} \end{array}$$

(6) $$\begin{array}{c} with\;\Delta P_{1}^{D4}=\Delta P_{2}^{D4}\cdot -1. \end{array}$$

It has to be assumed that only the physicians use the door D3 and D4 and the patients enter and leave through the same door. All cases in which *R* is bigger than two persons have to be checked, hence it is possible that a patient and an accompanying person enter the room separately and the physician enters later. It is inevitable that the specific analysis has to be adapted to each setting and the given circumstances.

There is also the possibility of installing a sensor at the main entrance door to the medical practice. This allows for evaluation of the total traffic, however, it has to be mentioned that the usage of these values is limited. With the current sensor system it is not possible to identify the person and so the calculation of the individual waiting time until the patient enters the physician’s room is not possible. More suitable is the comparison of the predicted appointment to the actual entering of the patient into the room. This evaluation can be used to optimise the appointment allocation. Further applications are evaluated in the following project phase for which five of the presented sensors systems are installed in different medical practices.

## 4. Discussion

As part of the initial desk model, different measurement methods were investigated. The tested ultrasonic range finders needed too large a spacing and the infrared light sensor was not as fast and accurate as needed. Both types are comparably large in relation to the time of flight (ToF) sensor. A further significant advantage of the ToF is that it can operate through a single small hole and has a very small observation footprint. In contrast to this, motion sensors based on the PIR technology are more suitable to observe a larger area/volume. Nevertheless, the chosen radar based MD allows independent verification of the motion detection, and in combination with the magnetic door switch, a workable combination was found.

In the literature, further detection approaches based on impulse radio ultra-wideband radar sensors are available. Choi et al. [29] showed that using this technique, a complete passage at a subway station in Seoul with a counting accuracy of less than 10% can be covered. Specific arrangements with WiFi signals can be also used to detect bi-directional motion [30,31]. Very promising is the expansion from a single point measurement to a scanning range [17,18], which also includes an increased effort in the post processing. Nevertheless, the inclusion of a further sensor type will be considered as part of the future work.

The distance of 10 cm between the two ToF sensors was chosen as similar to the commercially available products with infrared light sensors. Based on the prototype testing, the choice was proven functional and no further iteration was needed. A bigger distance would be desirable in principle. This would make the measurement clearer and would allow it to capture even faster movements. However, this would subsequently make it harder or even impossible to fit the device into the door frame, which increases the risk of the device being damaged or being a potential safety hazard. For locations, in which a mounting on the door frame is not possible, temporary boxes or other structures can be placed close to the door. It has to be ensured that the opposing side is rigid to avoid errors. Furthermore, the sensor is limited to 2 m and it is highly recommended not to make use of the full range. It should also be kept in mind that the area should be kept clear—objects such as umbrella stands or coat hooks can potentially trigger the measurement.

Two different heights of the ToF sensors were tested, namely 60 cm and 75 cm from the ground. Both installations showed good results but the lower one delivered slightly better results. The main concern is that the location is high enough to detect the body of the passing person but not too close to the doorknob, which could potentially lead to an incorrect measurement of a hand. For usage of this sensors in settings with mainly children the exact positioning should be evaluated.

The door contacts are at the moment only used opposite of the door hinges (Figure 1 and Figure 3). In this location the needed distance for releasing the magnetic contact is reached with a small opening angle. A mounting of the sensors on the same side as the hinges brings the advantage that the sensor is better protected as the persons are more likely to move closer to the doorknob through the door. This has to be tested as part of the further installation of the final version of the sensor.

The presented analysis is focused on the detection of one person passing through the door. It is not possible to detect the entrance of two people side by side or to distinguish between two people in direct physical contact at the height of the sensor system. The latter can occur for wheelchair users. Those cases can lead to a misinterpretation of the sensor readings if the supporting person leaves the room for the consultation. The involved physician will be informed of this and a manual correction will be needed, which can further be used to refine the analysis. Nevertheless, multiple persons can be registered separately if the time between them is larger than a defined threshold as shown in Figure 4c. The exact value for this threshold will be evaluated in the next step of the research project, however, delivered good results with the current setting. In the actual application this is more likely to be used in a multi-room setting in which the physician is switching between two rooms.

At state of the current project, each individual unit operates separately and no time module is included. Consequently, only a relative time beginning with the initialisation of the Arduino is available. The synchronisation is done manually, which is sufficient for the current applications. An additional time module would allow the exclusion of out-of-office time periods, in which the current sensors continues to observe. The data is currently stored only locally, which ensures that a potential interference with medical instruments is negligible but has the disadvantage that the operation cannot be controlled as long as the sensor is deployed. In a future step of the project, the final version of the sensor system will be installed in medical practices under authentic conditions. In the course of this, a direct comparison of the sensor system with an observation (similar to the stopwatch method) will be conducted. The recordings will be further checked with the total number of patients per day. Also, the acceptance and the need for a real time data transmission will be evaluated as well as the need for the integration of a battery.

## 5. Conclusions

Based on the specific requirements of healthcare applications, a novel sensor system was developed. The paper discusses the different investigated approaches which led to the final combination of three different types: (a) time of flight, (b) door sensor, and (c) motion detection. This combination allows the documentation of motion, including the direction of the person passing through the door, as well as a high reliability based on the different sensor types. The time resolution of the output has an accuracy of less than one second and allows the capture of the duration of the interaction between patient and physician. The limitation to one side of the door frame is an achieved goal and reduces the effort for the installation, as well as possible errors throughout a longer deployment, significantly.

The paper includes exemplary results based on the initial test and the developed concept for the quality control of the measurement. An expansion of the system for multiple doors, as well as rooms, is described. In a next step of the project, the sensor system is installed at different medical practices to measure the duration of patient–physician interactions. This will also include a direct comparison to an observation outside the door and also focus on possible problems with simultaneously entering persons (wheelchairs). The gained experiences will allow us to optimise the analysis and further reduce the needed manual work.

## Notation


*d*
distance sensor to opposing wall (mm)
${}_{i}$
index numbering of detected events at the door (-)
${}_{j}$
index numbering the loops (-)
$s_{1,2}$
measured value of the ToF sensor (mm)
Δs
difference (mm)
*t*
time (sec)
Δt
time step (sec)
ΔP
change (Person)
*R*
persons in the room (Person)DdoorDSdoor sensorGDPRGeneral Data Protection RegulationINRUDInternational Network for the Rational Use of DrugsMDmotion detectorPIRpassive infrared sensorToFTime of FlightWHOWorld Health Organisation

## Figures and Tables

**Table 1:** Decision matrix for time of flight measurement (ToF), door sensor (DS) and motion detector (MD), motion detection (mD), no detection (nD), opening (o) and closing (c) of the door, open (O) and closed (C) door. * Depending on the exact location of the sensor.

ToF	DS	MD	ΔP	Conclusion Event
mD	o + c	mD	±1	incoming or outgoing person
mD	O	mD	±1	incoming or outgoing person (open door)
mD	o + c	nD	±1	incoming or outgoing person (MD error)
mD	O	nD	±1	incoming or outgoing person (MD error)
mD	C	mD	0	passing by inside * or error
nD	O or C	mD	0	passing by outside * or error
nD	c	mD	0	door closed only (typical for automatic door closer)
nD	o	mD	0	door is opend but nobody comes in
nD	o + c	nD	0	error DS
